# The Role of Patient- and Treatment-Related Factors and Early Functional Imaging in Late Radiation-Induced Xerostomia in Oropharyngeal Cancer Patients

**DOI:** 10.3390/cancers13246296

**Published:** 2021-12-15

**Authors:** Simona Marzi, Alessia Farneti, Laura Marucci, Pasqualina D’Urso, Antonello Vidiri, Emma Gangemi, Giuseppe Sanguineti

**Affiliations:** 1 Medical Physics Laboratory, IRCCS Regina Elena National Cancer Institute, Via Elio Chianesi 53, 00144 Rome, Italy; simona.marzi@ifo.gov.it; 2 Department of Radiotherapy, IRCCS Regina Elena National Cancer Institute, Via Elio Chianesi 53, 00144 Rome, Italy; laura.marucci@ifo.gov.it (L.M.); pasqualina.durso@ifo.gov.it (P.D.); giuseppe.sanguineti@ifo.gov.it (G.S.); 3 Radiology and Diagnostic Imaging Department, IRCCS Regina Elena National Cancer Institute, Via Elio Chianesi 53, 00144 Rome, Italy; antonello.vidiri@ifo.gov.it (A.V.); emmagan86@gmail.com (E.G.)

**Keywords:** xerostomia, radiotherapy, parotid gland, oropharyngeal cancer, DWI, DCE-MRI

## Abstract

**Simple Summary:**

In the present prospective study, we assessed the role of various Magnetic Resonance Imaging biomarkers combined with self-assessed xerostomia questionnaires and patient- and treatment-related factors, in predicting xerostomia at 12 months after chemoradiotherapy for oropharyngeal squamous cell carcinoma. We hypothesized that the integration of pre-treatment imaging biomarkers, which addresses the tissue heterogeneity and individual variations among patients, could improve the accuracy of conventional prediction models that are based only on dose information, ultimately providing a better understanding of the pathophysiological mechanisms underlying radiation induced salivary dysfunction. The implementation of multifactorial models, driven by machine learning algorithms, may improve prediction accuracy of radiation-induced toxicity and tailor individual treatment options for patients.

**Abstract:**

The advent of quantitative imaging in personalized radiotherapy (RT) has offered the opportunity for a better understanding of individual variations in intrinsic radiosensitivity. We aimed to assess the role of magnetic resonance imaging (MRI) biomarkers, patient-related factors, and treatment-related factors in predicting xerostomia 12 months after RT (XER_12_) in patients affected by oropharyngeal squamous cell carcinoma (OSCC). Patients with locally advanced OSCC underwent diffusion-weighted imaging (DWI) and dynamic-contrast enhanced MRI at baseline; DWI was repeated at the 10th fraction of RT. The Radiation Therapy Oncology Group (RTOG) toxicity scale was used to evaluate salivary gland toxicity. Xerostomia-related questionnaires (XQs) were administered weekly during and after RT. RTOG toxicity ≥ grade 2 at XER_12_ was considered as endpoint to build prediction models. A Decision Tree classification learner was applied to build the prediction models following a five-fold cross-validation. Of the 89 patients enrolled, 63 were eligible for analysis. Thirty-six (57.1%) and 21 (33.3%) patients developed grade 1 and grade 2 XER_12_, respectively. Including only baseline variables, the model based on DCE-MRI and V65 (%) (volume of both glands receiving doses ≥ 65 Gy) had a fair accuracy (77%, 95% CI: 66.5–85.4%). The model based on V65 (%) and XQ-Int_mid_ (integral of acute XQ scores from the start to the middle of RT) reached the best accuracy (81%, 95% CI: 71–88.7%). In conclusion, non-invasive biomarkers from DCE-MRI, in combination with dosimetric variables and self-assessed acute XQ scores during treatment may help predict grade 2 XER_12_ with a fair to good accuracy.

## 1. Introduction

Xerostomia is one of the most prevalent side effects of RT (RT) for head and neck squamous cell carcinoma [1]. Persisting toxicity, even when non-life threatening, may appreciably impact the patient’s quality of life [2]. Despite technical improvements in RT planning and delivery, moderate to severe chronic xerostomia is observed in approximately 40% of patients undergoing intensity-modulated RT, due to technical limitations at restricting the dose to the parotids while adequately covering the primary tumour or the neck nodes [1,3,4].

Several studies have investigated the role of the average dose delivered to one or both parotids in predicting persistent dry mouth [5], finding a significant direct correlation between the planned dose and the severity of xerostomia following RT [6]. From these studies, a limiting mean dose of ≈25 Gy to both glands is advised to prevent chronic salivary dysfunction, which is rarely achieved considering the high doses (≈70 Gy) that are typically prescribed to the macroscopic disease [7].

Measurements of salivary output and scores from validated self-assessed xerostomia questionnaires (XQs) demonstrated to be useful for evaluating early and long side effects of RT and correlated with both submandibular and parotid dose [8].

More recently, a number of studies have investigated a variety of pre-treatment indices related to specific tissue signatures, such as fat concentration, cell density, and vascular perfusion, using either computed tomography (CT) or magnetic resonance imaging (MRI) [9,10]. These studies aimed to improve the accuracy of conventional prediction models based only on dosimetric variables [11], providing deeper insights into the complex mechanisms underlying radiation-induced salivary dysfunction [12].

The advent of quantitative imaging in personalized radiotherapy (RT) has offered the opportunity for a more comprehensive and non-invasive anatomical and functional characterization of tissues and for a better understanding of individual variations in intrinsic radiosensitivity [13,14]. Quantitative imaging also offers the opportunity to assess tissue heterogeneity and explore its association with different patterns of radiosensitivity within the organ. It was reported that different regions of the parotid gland could respond differently to radiation-induced damage and may have a different impact on xerostomia recovery [15]. In addition, it is still being investigated whether the mean dose and/or high doses delivered to a small portion of the gland could be harmful and have an impact on acute and late xerostomia [16]. 

All these investigational studies emphasized the importance of integrating image-based biomarkers of normal tissues with both patient- and treatment-related factors, in attempts to improve the limited predictive power of previous models of radiation-induced toxicity [17] and ultimately to personalize the patient management [13,14,15,18].

The aim of the present prospective study, funded by the Italian Association for Cancer Research (AIRC, project No. 17028), is to investigate the role of MRI biomarkers combined with self-assessed XQs and patient- and treatment-related factors, in predicting xerostomia at 12 months after chemoradiotherapy (XER_12_) for oropharyngeal squamous cell carcinoma (OSCC).

## 2. Materials and Methods

### 2.1. Patient Population and Treatment

After completing an informed consent form, patients were accrued into a prospective, single-institution cohort study approved by the local Institutional Review Board (approval number RS716/15).

The inclusion criteria were: age ≥ 18 years; pathologically confirmed squamous cell carcinoma of the oropharynx; and stage III or IV OSSC without distant metastases according to the 7th edition of AJCC [19]. The exclusion criteria were: any contraindication to MRI and/or chemoradiotherapy; previous surgery, chemotherapy or RT to the head and the neck; Zubrod performance status 2 or higher.

Patients received intensity-modulated RT (IMRT) and concomitant chemotherapy with cisplatin (100 mg/m^2^ for three cycles every 21 days or 40 mg/m^2^ weekly for 6 cycles). A dose of 70 Gy to areas of macroscopic disease, including primary tumour and pathologic lymph nodes, 63 Gy to regions at high risk of microscopic disease, and 58.1 Gy to regions at intermediate risk of microscopic disease was prescribed in 35 fractions [20]. A simultaneous integrated boost technique was applied using seven 6-MV photon beams.

The cumulative dose-volume histograms (DVHs) of each separate and combined parotid gland were extracted from the treatment planning system (Eclipse, Varian Medical System, Palo Alto, Santa Clara, CA, USA). T2-weighted images were also loaded in Eclipse and used for organ delineation (see Section 2.2). The DVHs were re-extracted from a second CT scan performed at the end of the second week of RT. The original treatment plan was transferred to the second CT scan, keeping the original beam configuration, fluence maps and monitor units. A rigid co-registration strategy between CT scans was applied using anatomical landmarks—typically C2—as a reference. Even though submandibular glands were not constrained during the planning process, the mean dose to both glands (D_mean,SMG_) at baseline was included in the analysis to explore its potential role to predict xerostomia.

### 2.2. MRI Protocol

MRI scans were acquired with a 1.5T system (Optima™ MR450w, GE Healthcare, Milwaukee, WI, United States) with a head and neck RF coil combination. Three serial scans were performed for each patient: at baseline, after the 10th fraction of RT, and 8 weeks after ceasing RT.

Before treatment, both intravoxel incoherent motion diffusion-weighted imaging (IVIM-DWI) and dynamic-contrast enhanced MRI (DCE-MRI) sequences were included in the protocol; however, only IVIM-DWI was performed at the 10th fraction to limit the use of contrast medium. In each scan, T2-weighted images (field of view, 26–28 cm; acquisition matrix, 288 × 256; slice thickness, 4 mm) were acquired in both coronal and axial planes. IVIM-DWI was performed using multiple *b* values (*b* = 0, 25, 50, 75, 100, 150, 300, 500, and 800 s/mm^2^, field of view 26 × 28 cm; acquisition matrix, 128 × 128; slice thickness, 4 mm; scanning time, 6 min 13 s). An optimized DCE-MRI based on a 3D fast-spoiled gradient echo sequence was acquired (flip angle, 30°; field of view, 28 cm; acquisition matrix, 128 × 128; slice thickness, 4 mm; spacing between slices, 2 mm; 60 dynamic phases; temporal resolution, 5 s; scanning time, 5 min). After three phases, a bolus of gadolinium-based contrast agent was intravenously administered at a rate of 3 mL/s.

Following RT, MRI was performed every 6 months for the first 2 years, and once per year thereafter.

### 2.3. Anatomical and Functional MRI Quantification

For image visualization and gland segmentation the free open source 3D Slicer Software (version 4.11) was used [21]. Each parotid gland was manually outlined on T2-weighted images acquired at baseline, at the 10th fraction, and at 8 weeks after treatment in order to estimate the relative (compared to baseline) organ shrinkage both during (ΔVol_10fr_) and after treatment (ΔVol_post_). Both superficial and deep lobes were included in the entire gland delineation.

After rigid propagation, the baseline contours were also used to perform quantitative analyses on perfusion maps. The parameters were: K^trans^, representing the transfer constant between plasma and the extravascular extracellular space (EES), and K_ep_, representing the transfer constant between EES and plasma and v_e_, which represents the fractional EES volume [22]. The model-free parameter IAUGC, defined as the initial area under the gadolinium concentration curve, was calculated from the bolus arrival to the first 90 s. In order to address the heterogeneity of organ vascularization and its potential association to xerostomia, the medians were analysed in combination with the percentiles (P) P10, P25, P75, and P90 and with the skewness, kurtosis, energy, and entropy values.

The perfusion-free tissue diffusion coefficient D*_t_* (in mm^2^/s) was derived from data at *b* values of 300, 500, and 800 s/mm^2^; whereas, the conventional ADC was derived from data at *b* values of 0, 500, and 800 s/mm^2^, using a mono-exponential function. The Levenberg–Marquardt algorithm was used to perform the fits.

Full details regarding the data extraction from DCE-MRI and DWI is reported in the Supplementary Materials.

### 2.4. Xerostomia Evaluation

The self-assessed xerostomia questionnaire (XQ) is a validated tool developed by Eisbruch et al. comprising eight questions aimed at evaluating different aspects and implications of xerostomia [23]. A score ranging from 0 (no symptoms) to 10 (most severe symptoms) is assigned to each answer. The XQ was administered at baseline, weekly during treatment, and at 3, 6-, 12-, 18-, and 24-months post-RT.

The curve of total XQ scores from all eight questions versus the week of treatment was plotted for each patient and fitted by a second-degree polynomial function to calculate summary indicators representative of the XQ score progress during treatment. Specifically, the gradient of the XQ score curve after one week of RT (XQ-Grad1), the gradient of the XQ score curve after four weeks of RT (XQ-Grad2), and the integral of the XQ score curve from the beginning to the middle of the treatment course, i.e., after the 15th fraction of RT, (XQ-Int_mid_) was extracted and included in the subsequent analyses. XQ-Grad1 and XQ-Grad2 indicate the rapidity of the XQ score increase in the first and second half of the RT course, respectively, whereas the integral XQ-Int_mid_ provides a measure of the cumulative XQ scores over the first half of the RT course.

The Radiation Therapy Oncology Group (RTOG) toxicity scale was used to score salivary gland toxicity [24]. The scale was applied prior to initiating RT, weekly during treatment, and at 3, 6, 12, 18, and 24 months after RT.

### 2.5. Statistics

The presence of RTOG toxicity grade ≥2 at 12 months after RT (XER_12_) was considered as an endpoint to build the prediction models. These models were developed considering both (1) only pre-treatment variables, and (2) both pre- and in-treatment variables.

After standardizing the dataset by the z-score normalization method [25], a Decision Tree classification learner was applied to build the prediction models. Considering the small sample size, a five-fold cross-validation was carried out to reduce the effect of overfitting [26]. The ADASYN (Adaptive Synthetic Sampling) algorithm was applied to improve group balance by synthetically creating new data from the minority class through linear interpolation between existing minority group data [27]. Only predictive models showing AUC > 0.6 were taken into consideration. To compare the prediction accuracies between models, the mid-*p*-value McNemar test was applied. Full description of the model building is available as Supplementary Materials.

A *p* level of <0.05 was considered statistically significant. All statistical analyses were performed in MATLAB (R2020b).

## 3. Results

This prospective study enrolled 89 patients between January 2016 and June 2019. Of the 89 patients, 18 died of progressive disease before the 12-month xerostomia assessment, and 8 declined to complete the follow-up questionnaires. The remaining cohort of 63 patients (126 parotids) were included in the study. Patient and tumour characteristics are summarized in Table 1. The case classified as unknown had a very small primary tumour, which was not visible at the diagnosis and was identified over the course of the subsequent workup as oropharyngeal squamous cell carcinoma.

All included patients received both RT and concomitant chemotherapy as planned, except for seven patients who did not receive chemotherapy. Patients undergoing chemotherapy did not show a different proportion of grade 2 XER_12_, compared to those not receiving chemotherapy (*p* = 0.12). The difference in chemotherapy schedules (3 cycles every 21 days versus 6 cycles per week) between patients with XER_12_ < grade 2 and XER_12_ = grade 2 was not significant (*p* = 0.76).

**Figure 1:**
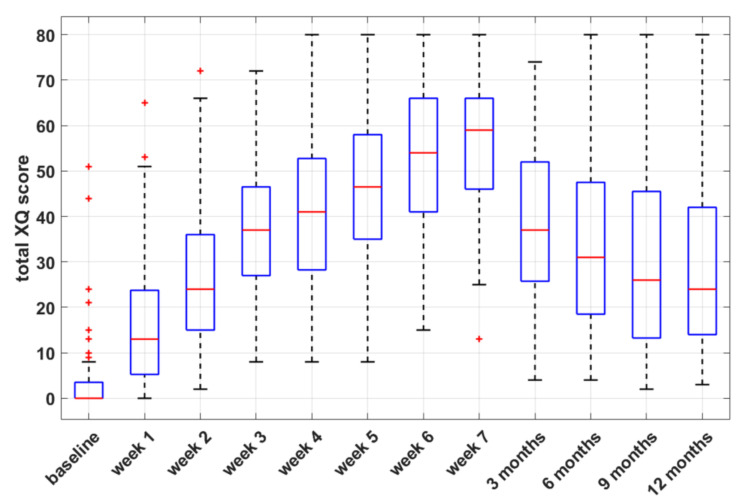
Box-and-whisker plots of self-assessed xerostomia-related questionnaire (XQ) scores versus time, during and after radiotherapy.

The prevalence rates of xerostomia at 12 months were 57.1% and 33.3% for grades 1 and 2, respectively. Of note, none of the patients with persisting toxicity had baseline xerostomia. The boxplots of self-assessed total XQ scores at selected time points during and after treatment (Figure 1) show a gradual decrease in XQ scores after RT, which is consistent with previous findings of partial relief of acute symptoms after RT [28].

The summary statistics of all selected imaging parameters, patient-related parameters (BMI, patient weight, parotid volume/shrinkage), and dosimetric data are reported in Tables S1–S12. Perfusion parameters were not available for four patients due to the inclusion of only a small portion of the parotids inside the volume covered within the DCE-MRI sequence.

### 3.1. Dose-Volume Points and Xerostomia

At baseline, several parotid dose-volume points differed significantly between patients with XER_12_ < grade 2 and XER_12_ = grade 2 (see Table S6 and Figure S1). The median (interquartile range; IQR) values of D_mean_ were 35.8 (6) Gy and 41.0 (7.8) Gy for patients with XER_12_ < grade 2 and XER_12_ = grade 2, respectively (*p* = 0.005). The volume percentage of both glands receiving doses ≥ 65 Gy (V65) were better able to differentiate between patients with and without toxicity (*p* < 0.001).

The second CT scan was acquired at fraction 11 ± 2.5. To derive parotid DVHs at the 10th fraction, DVH interpolation (between the baseline DVH and a second DVH re-evaluated after the 10th fraction) was applied in 15 patients, whereas DVH extrapolation (between the baseline DVH and a second DVH re-evaluated before the 10th fraction) was applied in six patients. D_mean_ at the 10th fraction increased slightly relatively to pre-treatment values (Wilcoxon test, *p* = 0.132), and the median (IQR) was 36.7 (8.2) Gy and 39.9 (9.3) Gy for patients with XER_12_ < grade 2 and XER_12_ = grade 2, respectively (*p* = 0.05). A number of dose variables derived at this time point significantly differentiated between patients with and without grade 2 XER_12_ but were not superior to pre-treatment ones (Table S7).

At baseline, the median (IQR) values of D_mean,SMG_ were 62.5 (4.6) Gy and 64.2 (3.1) Gy for patients with XER_12_ < grade 2 and XER_12_ = grade 2, respectively (*p* = 0.004).

### 3.2. Prediction Models of Xerostomia

The most significant predictors for grade 2 XER_12_ following univariate analysis are shown in Table 2. Box-and-whisker plots of the selected variables and the Spearman’s coefficient Rho between these variables are reported in Figure S2 and Table S12, respectively. Among categorical variables (sex, HPV status, tumour site, T stage, and N stage), only N stage was able to significantly discriminate between patients with XER_12_< and =grade 2 (Chi-squared *p* = 0.034, Table S11). However, N stage had a statistically significant impact on the dose to parotids, both D_mean_ and V65 (*p* = 0.038 and *p* = 0.02, respectively) and on the dose to submandibular glands D_mean,SMG_ (*p* = 0.008). Therefore, to reduce redundancy we did not incorporate N stage in the model building, including dosimetric variables related to both parotids and submandibular glands as alternatives.

Seven models incorporating only baseline variables and nine models incorporating variables assessed both at baseline and during treatment were obtained, whose predictive performances are reported in Table 3, respectively.

Taking into account only variables at baseline, the Model 3 based on P25 of v_e_ and V65 (%) had the highest receiver operating characteristic curve AUC (0.79) and accuracy (77%); when variables evaluated during treatment were also included, the Model 8 based on V65 (%) and XQ-Int_mid_ (the integral of acute XQ scores from the start to the middle of treatment) had the best AUC (0.80) and accuracy (81%). The predictive performance of this model (Model 8), although it includes only two variables, was comparable with those of Model 10 (*p* = 0.82), Model 12 (*p* = 1) and Model 13 (*p* = 0.84), which had similar accuracies but were based on three or four predictors. Model 8 did not show a significant difference relative to Model 3 (*p* = 0.86). The comparison between prediction accuracies of all the models is reported in Table S13. Models 5 and 6, based on D_mean,SMG,_ showed a significantly lower accuracies, compared to Model 3 (*p* = 0.04 in both comparisons) and Model 8 (*p* = 0.07 with a trend towards significance and *p* = 0.02, respectively).

### 3.3. Illustrative Cases

**Figure 2:**
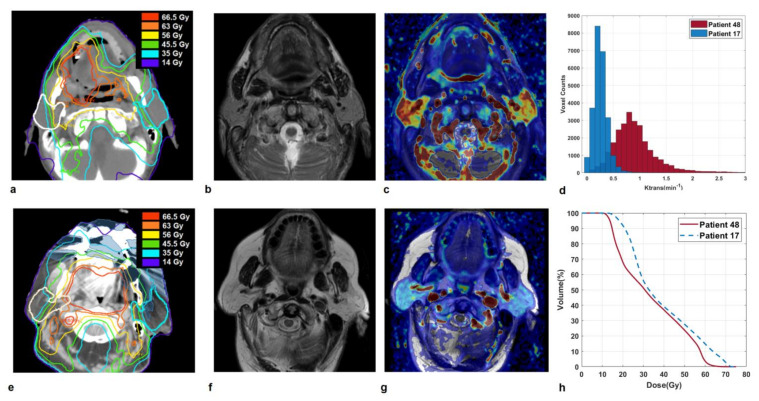
Sections of parotid glands in a 68-year-old man affected by a tonsil carcinoma (Patient 48) on axial CT image with overlaid isodose levels and parotid contours (**a**); the corresponding axial T2-weighted image (**b**) and map of K^trans^ (**c**) which indicates highly vascularized glands. Analogously, sections of parotid glands in a 75-year-old woman affected by a soft palate carcinoma (Patient 17) on axial CT image with overlaid isodose levels and parotid contours (**e**); the corresponding axial T2-weighted image (**f**) and map of K^trans^ (**g**) which shows moderately perfused glands. K^trans^ histograms (**d**) and dose-volume histograms (**h**) of both parotids, for Patient 48 and 17 in comparison. Patient 48, who developed grade 1 XER_12_, had much more vascularized parotids glands than Patient 17, who experienced grade 2 XER_12_ (median K^trans^ = 0.93 versus 0.19 min^−1^). He received little lower doses to parotids (D_mean_/V65 = 33.3 Gy/0.4% versus 38.1 Gy/8.5%) but showed much lower in-treatment XQ scores, compared to patient 17 (XQ-Int_mid_ = 33.2 versus 82.9, respectively).

**Figure 3:**
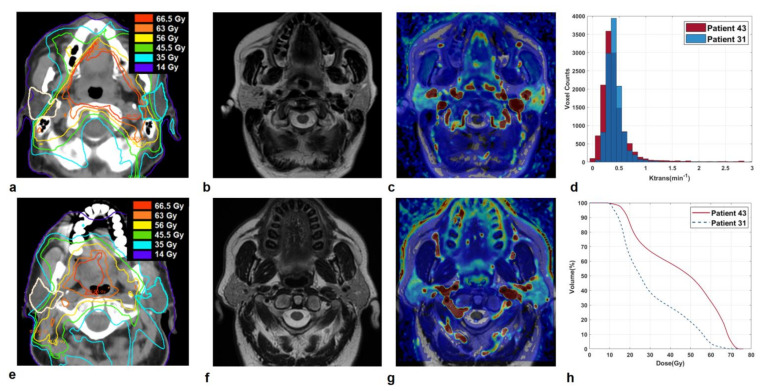
Sections of parotid glands in a 47-year-old woman affected by a base of the tongue carcinoma (Patient 43) on axial CT image with overlaid isodose levels and parotid contours (**a**); the corresponding axial T2-weighted image (**b**) and map of K^trans^ (**c**). Analogously, sections of parotid glands in a 69-year-old man affected by a base of the tongue carcinoma (Patient 31) on axial CT image with overlaid isodose levels and parotid contours (**e**); the corresponding axial T2-weighted image (**f**) and map of K^trans^ (**g**). K^trans^ histograms (**d**) and dose-volume histograms (**h**) of both parotids, in comparison.

Some illustrative cases are shown in Figure 2 and Figure 3, where imaging and parotid dose-volume histograms of patients experiencing grade 1 and grade 2 XER_12_ are displayed in comparison. In Figure 2, the dose delivered to parotids was similar and the disparity in late xerostomia may be attributed to the differences in gland perfusion at baseline and to the gap in acute XQ scores during treatment (see Figure S3). Conversely, the two patients in Figure 3 had comparable parotid gland perfusion, but received very different doses to parotids, which led to a large disparity in acute XQ scores and, as a result, to a disparity in late xerostomia.

Patient 31 and patient 43, who experienced grade 1 and grade 2 XER_12_, respectively, had parotids glands with quite similar perfusion levels (median K^trans^ = 0.36 versus 0.40 min^−1^). However, they received very different doses to parotid glands: D_mean_/V65 was 30.2 Gy/1.2% versus 44.7 Gy/21.2%, respectively. As a result, Patient 43 with grade 2 XER_12_ showed higher in-treatment XQ scores, compared to patient 31 (XQ-Int_mid_ = 127 versus 42, respectively), which led to a disparity in late toxicity.

## 4. Discussion

In the present study, we hypothesized that the integration of pre-treatment functional MRI biomarkers with self-assessed XQ scores, and dosimetric- and patient-related factors could improve the accuracy of conventional prediction models that are based only on dose information [11], simultaneously providing a better description of the pathophysiological mechanisms underlying radiation-induced salivary dysfunction [10,12,15,29].

After some exploratory tests to quickly train the most common machine learning algorithms [30], we found that, on our dataset, the Decision Tree classifier provided comparable or better accuracies in most of the different combination of selected predictors. Sixteen different models were obtained that included either (1) only pre-treatment variables or (2) pre-treatment variables and variables determined during treatment. The first class of models confirmed the significant role of the planned mean parotid dose, as already demonstrated by several studies [15], but also emphasized the detrimental effect of the high doses delivered to small gland parts, specifically through the parameter V65 (%) at baseline. These results are consistent with a recent study reporting that a high dose delivered to a small gland sub-volume may have a more detrimental impact on injury than a low-dose bath [16], whereas the addition of D_mean,SMG_ did not significantly improve the predictive performance of the models, compared to those including only parotid dosimetric variables. This may be due to the fact that, despite the significant differences found in D_mean,SMG_ between patients with XER_12_< and =grade 2, submandibular glands received doses well above the mean dose threshold of <39 Gy, which has been suggested to limit radiation-induced damage [8]. In contrast, the partial recovery of parotid glands functionality after treatment may have contributed to their central role in predicting late xerostomia [28].

It should be noted that models including fewer predictors had comparable or better performances than more complex models based on a larger number of predictors; in fact, the selection of a lower number of variables helps reduce redundancy and irrelevance, and to avoid the problem of overfitting [30].

Because the planned and actual parotid dose may differ due to anatomical modifications of the patient throughout the course of RT [31,32], we re-evaluated DVHs from a second CT scan acquired at the 10th fraction to quantify this potential confounding factor. Even though some dose variables at this time point were significantly related to grade 2 XER_12_, they were not superior to pre-treatment ones and were not selected for the model building.

Several parameters derived from DCE-MRI maps were useful for predicting grade 2 XER_12_. The histogram-based approach and the extraction of percentiles increased the ability to find associations between DCE-MRI parameters and xerostomia, compared to more simplistic mean values. Notably, patients with hypoperfused parotids were found to be more likely to experience greater toxicity. Our findings are indirectly supported by the study of van Dijk et al., who investigated the relationship between late xerostomia and fatty parotid components at MRI and found a direct relationship between signal intensity (P90) from T1-weigthed MRI and XER_12_ [8]. Concurrently, a negative correlation between gland vascularization and BMI [33] and a positive association between BMI and parotid fat content in healthy adults have been documented [34]. BMI may also partly explain why patients with grade 2 XER_12_ had lower v_e_ values, compared to patients with grade <2 XER_12_, considering that a larger fatty component may cause a reduction in the EES.

In-treatment parameters may contain important information on individual responses to treatment—particularly in week 3—thereby allowing for treatment personalization [35]. This was confirmed by the second class of models that we proposed, which incorporated in-treatment variables. In fact, Model 8, which was based on both V65 (%) and XQ-Int_mid_, had the best AUC (0.80) and accuracy (0.81) and highlighted the importance of self-assessed acute XQ scores in predicting late xerostomia. As mentioned, patients may perceive dry mouth differently, despite their salivary production [5], and observer-based grades may differ from the patients’ subjective scores [8,36]. 

The AUCs of our long-term xerostomia prediction models were similar to those reported in the recent literature based on CT, PET, or MR imaging biomarkers [15], despite the fact that a direct comparison was challenging due to differences in the nature of the investigation (retrospective vs. prospective), follow-up time, patient-specific and dose factors studied, patient population, and statistical methods used.

Presently, the relationship between radiation-induced parotid atrophy and late xerostomia remains controversial; some studies indicate a correlation between the gland shrinkage and the degree of reduction in saliva production after RT [37], while others show that patients with less shrinkage at mid-treatment were more likely to experience a worse outcome [38]. Based on our data, the volumes of parotids at each time point (at baseline, at 10th fraction, at 8 weeks post-RT) and their changes relative to baseline did not significantly differ between the two patient groups, even though patients with smaller parotid glands, particularly at the 10th fraction, were more likely to experience grade 2 XER_12_ with a trend toward statistical significance [11]. 

It has been reported that early quantification of parotid density variations, based on Hounsfield Units (HU) measurements from CT scans, could represent more robust and sensitive biomarkers of acinar cell reduction or acute xerostomia, instead of volume shrinkage [39,40]. Similarly, we assumed that early variations in ADC, and D_t_ in particular—which is a perfusion-free diffusion coefficient [41]—could be more strongly related to modifications in cellular microstructure and cell death than to volume changes, as previously suggested [42]. However, neither ADC nor D_t_—nor their variations during treatment—differed significantly between patients with XER_12_ < grade 2 and XER_12_ = grade 2. At the same time, our data indicated that both diffusion coefficients increased significantly during treatment, and their variations were strongly associated with D_mean_ (Gy) for several dose-volume points (see the Supplementary Materials), according to previous literature [33,43,44].

The study has some limitations. The performance of the models may be underestimated by the fact that they were evaluated on the internal test set after a stratified 5-fold cross-validation, which may not be representative of the entire data set due to the small sample size. We did not include ADC/D_t_ histogram analysis, though it was suggested by others [45] that early changes in the highest ADC percentiles correlated to Grade 2 xerostomia and could be clinically useful. Future investigations will include the role of D_t_ histogram analysis in particular, with the aim of improving the sensitivity of the classification model. Moreover, our study did not investigate the radiation effect in relation to the local dose delivery and to the involvement of specific regions of the gland that may be more radiosensitive [28,46]. Nevertheless, the incorporation of voxel-dose parameters does not appear to significantly improve the model’s predictive ability relative to conventional dosimetric parameters [28]. Moreover, Sari et al. recently reported that that D_mean_ to parotid stem cells (located inside the Stenson duct perimeter) had a comparable ability to predict xerostomia than did D_mean_ to entire parotids [47]. Lastly, the performance of the proposed models was not validated in an independent external cohort, which would have allowed them to demonstrate their robustness and transportability to other settings.

## 5. Conclusions

Non-invasive MRI biomarkers from DCE-MRI, together with dosimetric variables (both at intermediate and high doses) and self-assessed acute XQ scores during treatment, were significantly associated with xerostomia and may help predict grade 2 XER_12_ with a fair to good accuracy. These findings may aid physicians in the identification of specific risk factors and, consequently, improve patient management.

## Figures and Tables

**Table 1:** Selected patient and tumour characteristics.

Characteristic	Parameter	
Patient number	63	
Sex (M/F)	50 (79%)/13 (21%)	
Age (years)		
Mean (range)	66.6 (48–86)	
HPV status (−/+)	19 (30%)/44 (70%)	
Primary tumour site		
Tonsil	32 (50.8%)	
Base of Tongue	29 (46.0%)	
Soft Palate	1 (1.6%)	
Unknown	1 (1.6%)	
T stage		
	HPV−	HPV+
T0	0	1
T1	3	4
T2	5	16
T3	2	6
T4	0	17
T4a	9	0
N stage		
	HPV−	HPV+
N0	3	4
N1	3	17
N2	6	23
N3	7	0

**Table 2:** Selected predictors for Xerostomia Grade ≥2 after 12 months of RT.

	XER_12_ < 2		XER_12_ = 2		
	Median	IQR	Median	IQR	*p* Value
*K^trans^ P10 (min^−1^)*	0.28	0.20	0.21	0.13	0.026
*v_e_ P25*	0.15	0.08	0.13	0.05	0.049
*D_mean_ (Gy)*	35.8	6.0	41.0	7.8	0.004
*V65(%)*	6.5	8.6	10.1	12.3	<0.001
*D_mean,SMG_ (Gy)*	62.5	4.6	64.2	3.13	0.004
*XQ-Int_mid_*	51.5	41.5	82.9	78.4	0.023

Abbreviations: RTOG, Radiation Therapy Oncology Group; P10/P25, 10th/25th percentiles; K^trans^, transfer constant between plasma and extravascular extracellular space (EES) (min^−1^); v_e_, fractional volume of EES (fractional units); pre-treatment D_mean_ to both parotid glands; V_65_ (%), percentage of parotid volume receiving a dose ≥ 65 Gy; pre-treatment D_mean,SMG_ to both submandibular glands; XQ-Int_mid_ = integral of acute XQ scores acute xerostomia-related questionnaire scores from the start to mid treatment time (dimensional); *p* values refer to Mann–Whitney test.

**Table 3:** Predictive Performance of the models including variables at baseline.

Variables at Baseline		AUC *	Accuracy (%)	Sensitivity (%)	Specificity (%)	PPV (%)	NPV (%)
Model 1	*v_e_ P25* *D_mean_*	0.68	68.3	66.7	69.1	51.5	80.8
		[0.57–0.77]	[57.2–78.0]	[50.5–80.4]	[52.9–82.4]	[39.2–63.6]	[72.4–87.1]
Model 2	*K^trans^ P10 D_mean_*	0.61	59.5	64.3	57.1	42.5	76.5
		[0.50–0.70]	[48.2–70.1]	[48.0–78.5]	[41.0–72.3]	[32.8–52.8]	[66.7–84.0]
Model 3	*v_e_ P25* *V65(%)*	0.79	77.0	83.3	73.8	61.1	90.0
		[0.69–0.86]	[66.5–85.4]	[68.6–93.0]	[58.0–86.1]	[48.1–72.6]	[81.7–94.8]
Model 4	*K^trans^ P10 V65(%)*	0.70	67.4	78.6	61.9	50.4	85.4
		[0.60–0.80]	[56.3–77.2]	[63.2–89.7]	[45.6–76.4]	[40.1–60.6]	[75.8–91.6]
Model 5	*v_e_ P25* *D_mean_, _SMG_*	0.67	64.2	73.8	59.5	47.3	82.2
		[0.56–0.76]	[53.0–74.4]	[58.0–86.1]	[43.3–74.4]	[37.4–57.5]	[72.4–89.0]
Model 6	*K^trans^ P10 D_mean_, _SMG_*	0.64	65.1	61.9	66.7	47.8	78.0
		[0.53–0.74]	[53.9–75.2]	[45.6–76.4]	[50.5–80.4]	[35.9–59.9]	[69.6–84.7]
Model 7	*v_e_ P25* *V65(%)* *D_mean_, _SMG_*	0.71	71.4	71.4	71.4	55.2	83.5
		[0.63–0.81]	[60.5–80.8]	[55.4–84.3]	[55.4–84.3]	[42.4–67.3]	[75.2–89.5]
**Variables at Baseline and during RT**		AUC *	**Accuracy (%)**	**Sensitivity (%)**	**Specificity (%)**	**PPV (%)**	**NPV (%)**
Model 8	*V65(%)* *XQ-Int_mid_*	0.80	81.0	76.2	83.3	69.3	87.7
		[0.71–0.88]	[71.0–88.7]	[60.6–88.0]	[68.6–93.0]	[52.9–81.9]	[80.3–92.5]
Model 9	*D_mean_* *XQ-Int_mid_*	0.63	61.1	69.1	57.1	44.2	78.9
		[0.52–0.72]	[49.8–71.5]	[52.9–82.4]	[41.0–72.3]	[34.6–54.3]	[69.0–86.3]
Model 10	*v_e_ P25* *V65(%)* *XQ-Int_mid_*	0.79	78.6	78.6	78.6	64.4	88.2
		[0.69–0.86]	[68.3–86.8]	[63.2–89.7]	[63.2–89.7]	[49.8–76.7]	[80.3–93.1]
Model 11	*K^trans^ P10 V65(%)* *XQ-Int_mid_*	0.73	72.2	73.8	71.4	56.3	84.5
		[0.63–0.81]	[61.4–81.4]	[58.0–86.1]	[55.4–84.3]	[43.6–68.3]	[76.1–90.4]
Model 12	*v_e_ P25* *K^trans^ P10 V65(%)* *XQ-Int_mid_*	0.80	77.8	85.7	73.8	62.0	91.2
		[0.70–0.87]	[67.4–86.1]	[71.5–94.6]	[58.0–86.1]	[49.2–73.4]	[82.8–95.7]
Model 13	*v_e_ P25* *V65(%)* *D_mean_, _SMG_* *XQ-Int_mid_*	0.79	78.6	78.6	78.6	64.7	88.0
		[0.69–0.87]	[68.3–86.8]	[63.2–89.7]	[63.2–89.7]	[50.1–76.9]	[80.1–93.1]
Model 14	*v_e_ P25* *K^trans^ P10 D_mean_* *XQ-Int_mid_*	0.63	60.3	71.4	54.8	44.1	79.3
		[0.53–0.72]	[49.1–70.8]	[55.4–84.3]	[38.7–70.2]	[34.9–53.6]	[68.9–87.0]
Model 15	*v_e_ P25* *K^trans^ P10 D_mean_* *V65(%)* *XQ-Int_mid_*	0.71	73.0	66.7	76.2	58.3	82.1
		[0.61–0.81]	[62.2–82.1]	[50.5–80.4]	[60.6–88.0]	[43.9–71.4]	[74.3–87.9]
Model 16	*v_e_ P25* *K^trans^ P10 D_mean_* *V65(%)* *D_mean_, _SMG_* *XQ-Int_mid_*	0.74	71.4	81.0	66.7	54.8	87.5
		[0.63–0.83]	[60.5–80.8]	[65.9–91.4]	[50.5–80.4]	[43.6–65.6]	[78.4–93.1]

* Confidence Interval for the model AUC estimates calculated with bias corrected and accelerated percentile bootstrap method; the prediction model performance was estimated on the internal test set after a stratified 5-fold cross-validation. In squared brackets the 95% confidence interval is reported. Abbreviations as in Table 2.

## Data Availability

The original contributions presented in the study are included in the article/supplementary material. Further inquiries can be directed to the corresponding author.

## Supplementary Materials

The following are available online at https://www.mdpi.com/article/10.3390/cancers13246296/s1, Materials and Method: DCE-MRI and DWI Quantification; Statistical tests and model building, Table S1: DCE-MRI parameters sorted by RTOG classification, Table S2: DWI parameters sorted by RTOG classification, Table S3: Body mass index (BMI) and patient weight sorted by RTOG classification, Table S4: Volume of both glands. as single organ. sorted by RTOG classification, Table S5: Quantitative parameters derived from Acute Xerostomia-related Questionnaire (XQ) scores sorted by RTOG classification, Table S6 Dose–volume data of both glands. as single organ at baseline. sorted by RTOG classification, Table S7: Dose–volume data of both glands. as single organ at 10th fraction. sorted by RTOG classification, Table S8: Summary statistics of the diffusion parameters and their variations during treatment, Table S9: Spearman’s Rho values between ADC/Dt variations and dose-volume data of both glands, as single organ at baseline, Table S10: Spearman’s Rho values between ADC/Dt variations and dose-volume data of both glands at 10th fraction, Table S11: Chi—squared test between Nstage and XER12, Table S12 Spearman’s coefficient Rho between selected (continuous) variables for XER12 prediction model, Table S13: Comparison between prediction accuracies of the models, Figure S1: The average of the distribution of cumulative dose–volume histograms (DVHs) of both parotids, Figure S2: Box-and-whisker plots of the most significant predictors for XER12 = grade 2, Figure S3. Plots of acute XQ-score versus week of RT in two representative cases.
