# Supplementary material for: The Role of Patient- and Treatment-Related Factors and Early Functional Imaging in Late Radiation-Induced Xerostomia in Oropharyngeal Cancer Patients
Source: Cancers (Basel). 2021 Dec 15;13(24):6296. doi: 10.3390/cancers13246296 (PMC8699504; doi:10.3390/cancers13246296)
Supplement: Supplementary file 1 [file cancers-13-06296-s001.zip › cancers-1464871-supplementary.pdf]

cancers

MDPI

Materials and Method

DCE-MRI and DWI Quantification

A commercial software (GenIQ General, GE Advanced Workstation, Palo Alto, CA) was used to extract the maps from the DCE-MRI data, based on a two-compartment pharmacokinetic model. The medians, percentiles, skewness, kurtosis, energy, and entropy values were calculated from the voxel-based distribution of perfusion parameters within the three central slices of the gland. In fact, DCE-MRI did not include the entire organ in some patients due to the limited volume coverage, which was primarily optimized in relation to the tumor location. The bin size used for each patient to extract the data was 0.05 min^{-1}, 0.2 min^{-1}, 0.02 and 0.05 for K^{trans}, K_{ep}, v_e and IAUGC, respectively.

The parotids at baseline and at the 10th fraction were outlined on DWI at $b = 800$ s/mm^2 to extract the diffusion coefficients from the signal intensity curve at increasing b values, by means of home-made scripts developed in MATLAB (Release 2020b, MathWorks Inc., Natick, MA). To reduce the instability of the diffusion-weighted signal within single voxels and increase the robustness of ADC and D_t quantification, the median value derived from the entire gland was calculated for each b value and used for the fitting.

We decided not to include the fractional volume of capillary blood (f) and the perfusion-related diffusion coefficient (D*) in the present study, as no clear evidence of the capability of IVIM-DWI in quantifying tissue vascularization have been documented to date [41].

Statistical tests and model building

The relationships between categorical variables and the classification endpoint were estimated using the chi-squared test. The Kruskal–Wallis test was used to determine whether a given categorical variable had a statistically significant influence on a given continuous variable.

The Mann–Whitney U test was performed for the selection of the most significant continuous predictors for XER_{12}, using a cutoff p value of 0.10. In case of redundant variables or variables with a Spearman's correlation coefficient (ϱ) >0.8, the variable with the strongest association with XER_{12} was selected for the analysis.

A Decision Tree classification learner was applied to build the prediction models. The maximum number of split to control the depth of the tree was 20 and the Gini's diversity index was used as split criterion. The confidence interval for the area under the receiver operating characteristic curve (AUC) of each model was calculated with bias-corrected and accelerated percentile bootstrap method, using 1000 replicates. The model classification ability was also evaluated in terms of accuracy, sensitivity, specificity, positive predictive value (PPV), and negative predictive value (NPV) on the internal test set, after a stratified 5-fold cross-validation; this was done to correct for any optimism when evaluating the model's performance [24].

Table S1. DCE-MRI parameters sorted by RTOG classification.

		**XER_{12} < grade 2**		**XER_{12} = grade 2**		
		Median	*IQR*	*Median*	*IQR*	*P value**
	Median	0.42	0.36	0.34	0.21	0.094
	IQR	0.15	0.14	0.13	0.14	0.347
	P10	0.28	0.20	0.21	0.13	**0.026**
K^{trans}	*P25*	0.35	0.25	0.28	0.15	0.056
	P75	0.50	0.44	0.44	0.31	0.134
	P90	0.61	0.59	0.52	0.45	0.164
	Skewness	2.74	3.49	3.49	3.39	0.876

	Kurtosis	20.46	36.20	22.84	54.15	0.915
	Mean	0.45	0.41	0.38	0.30	0.126
	Std	0.24	0.23	0.16	0.24	0.529
	Energy	0.42	0.26	0.49	0.30	0.428
	Entropy	1.58	1.06	1.36	1.15	0.355
	Median	2.76	1.20	2.38	0.88	0.291
	IQR	0.96	1.16	0.90	0.78	0.647
	P10	1.68	0.96	1.42	0.56	0.259
	P25	2.12	0.96	1.92	0.52	0.306
	P75	3.36	1.68	2.80	1.21	0.427
K_{ep}	*P90*	3.99	2.52	3.56	1.68	0.386
	Skewness	2.98	3.70	2.58	2.90	0.139
	Kurtosis	22.79	41.62	19.83	29.92	0.162
	Mean	2.98	1.51	2.39	1.00	0.314
	Std	1.34	1.08	0.82	1.12	0.457
	Energy	0.36	0.25	0.38	0.19	0.689
	Entropy	1.83	1.07	1.67	0.92	0.641
	Median	0.17	0.10	0.15	0.06	0.055
	IQR	0.05	0.03	0.05	0.02	0.193
	P10	0.13	0.08	0.11	0.05	0.057
	P25	0.15	0.08	0.13	0.05	**0.049**
	P75	0.19	0.11	0.17	0.06	0.054
v_e	*P90*	0.22	0.14	0.20	0.09	0.100
	Skewness	1.31	1.58	1.83	3.34	0.256
	Kurtosis	8.29	8.78	11.89	28.50	0.193
	Mean	0.18	0.10	0.15	0.06	0.061
	Std	0.06	0.04	0.04	0.04	0.390
	Energy	0.19	0.13	0.20	0.09	0.177
	Entropy	2.79	1.04	2.73	0.69	0.249
	Median	0.26	0.15	0.20	0.09	**0.039**
	IQR	0.07	0.04	0.07	0.04	0.390
	P10	0.18	0.11	0.15	0.07	**0.030**
	P25	0.22	0.13	0.17	0.07	**0.034**
	P75	0.29	0.17	0.25	0.11	**0.049**
IAUGC	*P90*	0.34	0.22	0.29	0.13	0.061
	Skewness	1.40	1.11	1.32	1.27	0.618
	Kurtosis	8.77	7.28	7.66	5.10	0.762
	Mean	0.26	0.16	0.22	0.10	**0.043**
	Std	0.07	0.05	0.06	0.04	0.291
	Energy	0.21	0.14	0.23	0.12	0.540
	Entropy	2.65	0.96	2.50	0.80	0.307

P values refer to Mann-Whitney test*. Statistically significant p-values are ***bold.***

Table S2. DWI parameters sorted by RTOG classification.

	**XER_{12} < grade 2**		**XER_{12} = grade 2**		
	Median	*IQR*	*Median*	*IQR*	*P value*
ADC	1.25	0.17	1.24	0.24	0.625
D_t	0.94	0.17	0.97	0.22	0.699
ADC_{10fr}	1.46	0.21	1.50	0.25	0.128
$D_{t,10fr}$	1.08	0.22	1.10	0.12	0.237
ADC(%)	14.85	17.67	15.48	21.91	0.453

D_t(%)	15.00	22.41	16.31	21.18	0.756

P values refer to Mann-Whitney test*.

Table S3. Body mass index (BMI) and patient weight sorted by RTOG classification.

	**XER_{12} < grade 2**		**XER_{12} = grade 2**		
	Median	*IQR*	*Median*	*IQR*	*P value*
**BMI(Kg/m^2)**	24.30	4.05	25.34	4.46	0.218
**BMI$_{10fr}$(Kg/m^2)**	23.72	3.37	25.55	5.41	0.144
**BMI$_{end\ RT}$(Kg/m^2)**	21.76	5.60	22.69	4.31	0.252
**BMI$_{10fr}$(%)**	-1.82	4.17	-1.44	4.34	0.402
**BMI$_{10f}$(Kg/m^2)**	-0.43	1.08	-0.33	1.26	0.433
**BMI$_{end\ RT}$(%)**	-10.53	8.35	-8.64	9.89	0.485
**BMI$_{end\ RT}$(Kg/m^2)**	-2.47	2.27	-2.30	3.00	0.800
**Weight(Kg)**	74.00	14.50	75.00	17.25	0.971
**Weight$_{10fr}$(Kg)**	72.75	14.50	74.75	17.00	0.712
**Weight$_{end\ RT}$(Kg)**	66.00	14.75	68.50	16.38	0.802
**Weight$_{10fr}$(%)**	-1.82	4.17	-1.46	4.61	0.437
**Weight$_{10fr}$**	-1.25	3.50	-1.00	3.25	0.352
**Weight$_{end\ RT}$(%)**	-10.47	7.93	-8.64	9.01	0.671
**Weight$_{end\ RT}$(Kg)**	-7.50	7.00	-7.00	8.38	0.814

P values refer to Mann-Whitney test*.

Table S4 Volume of both glands. as single organ. sorted by RTOG classification.

	**XER_{12} < grade 2**		**XER_{12} = grade 2**		
	Median	*IQR*	*Median*	*IQR*	*P value*
Vol (cm^3)	65.3	25.0	55.4	28.3	0.168
Vol $_{10fr}$ (cm^3)	55.3	21.5	45.7	22.0	0.120
Vol$_{post}$(cm^3)	39.4	19.8	35.2	15.9	0.155
Vol $_{10fr}$ (%)	14.7	12.4	16.5	14.5	0.585
Vol$_{10fr}$ (cm^3)	8.6	7.0	8.3	7.7	0.896
Vol $_{post}$ (%)	35.3	21.1	26.1	33.7	0.489
Vol $_{post}$ (cm^3)	19.2	20.2	14.9	21.0	0.155

P values refer to Mann-Whitney test*.

Table S5. Quantitative parameters derived from Acute Xerostomia-related Questionnaire (XQ) scores sorted by RTOG classification.

	**XER_{12} < grade 2**		**XER_{12} = grade 2**		
	Median	*IQR*	*Median*	*IQR*	*P value*
XQ-Grad1	10.14	6.06	13.0	8.54	0.17
XQ-Grad2	6.00	4.51	6.71	4.51	0.624
XQ-Int$_{mid}$	51.5	41.50	82.9	78.4	**0.023**

XQ-Grad1= gradient of the XQ score curve after one week of RT; XQ-Grad2 = gradient of the XQ score curve after four weeks of RT; XQ-Int$_{mid}$ = the integral of the XQ score curve from the start to mid treatment. Statistically significant p-values are ***bold.***

Table S6. Dose-volume data of both glands. as single organ at baseline. sorted by RTOG classification.

	**XER_{12} < grade 2**		**XER_{12} = grade 2**		
	Median	*IQR*	*Median*	*IQR*	*P value*

V20(%)	75.9	19.2	80.2	16.2	0.177
V25(%)	63.9	17.2	69.2	15.3	0.089
V30(%)	54.4	12.1	62.2	15.1	**0.038**
V35(%)	46.8	15.5	57.3	15.5	**0.015**
V40(%)	40.9	14.8	50.8	16.1	**0.015**
V45(%)	34.9	11.4	45.0	17.0	**0.016**
V50(%)	28.3	10.0	36.8	19.4	**0.013**
V55(%)	21.4	8.9	28.2	20.7	**0.008**
V60(%)	13.0	10.7	18.4	16.5	**0.001**
V65(%)	6.5	8.6	10.1	12.3	**0.000**
V70(%)	1.0	2.3	3.4	3.9	**0.000**
D_{mean}(Gy)	35.8	6.0	41.0	7.8	**0.005**
H-D_{mean} (Gy)	38.5	7.0	43.2	10.2	**0.004**

H-D_{mean} is the highest mean dose between left and right parotid. Statistically significant p-values are ***bold.***

Table S7. Dose-volume data of both glands. as single organ at 10th fraction. sorted by RTOG classification.

	**XER_{12} < grade 2**		**XER_{12} = grade 2**		
	Median	*IQR*	*Median*	*IQR*	*P value*
V20(%)	74.1	19.1	79.2	17.1	0.182
V25(%)	65.4	18.6	69.2	17.5	0.235
V30(%)	55.5	14.5	63.3	18.2	0.151
V35(%)	49.0	17.7	55.3	18.4	0.095
V40(%)	43.6	17.3	49.4	18.9	0.079
V45(%)	37.7	15.6	44.9	18.6	0.061
V50(%)	31.2	12.8	35.7	21.3	0.092
V55(%)	24.2	11.0	28.0	23.0	0.061
V60(%)	14.7	8.5	18.5	16.2	**0.020**
V65(%)	7.1	10.6	10.9	11.5	**0.019**
V70(%)	2.1	4.2	4.3	5.7	**0.015**
D_{mean}(Gy)	36.7	8.2	39.9	9.3	**0.050**
H-D_{mean} (Gy)	38.8	9.8	43.2	10.5	**0.039**

H-D_{mean} is the highest mean dose between left and right parotid. Statistically significant p-values are ***bold***.

Table S8. Summary statistics of the diffusion parameters and their variations during treatment.

**Parameter***	**before RT**	**at the 10th**	**Variation from baseline (%)**	**p**
ADC (10^{-3} mm^2/s)	1.25 (0.17)	1.46 (0.21)	14.9 (17.7)	**<0.0001**
D_t (10^{-3} mm^2/s)	0.94 (0.17)	1.08 (0.22)	15.0 (22.4)	**<0.0001**

Data are reported as median(IQR). P values refer to Wilcoxon test. Statistically significant p-values are ***bold***.

Table S9. Spearman's Rho values between ADC/Dt variations and dose-volume data of both glands, as single organ at baseline.

	**ADC(%)**		**D_t (%)**	
	**Spearman's Rho**	**p-value**	**Spearman's Rho**	**p-value**

V20(%)	0.053	0.687	-0.041	0.758
V25(%)	0.172	0.190	0.097	0.459
V30(%)	0.341	0.008	0.277	**0.032**
V35(%)	0.411	**0.001**	0.362	**0.004**
V40(%)	0.465	**0.000**	0.434	**0.001**
V45(%)	0.468	**0.000**	0.451	**0.000**
V50(%)	0.438	**0.000**	0.435	**0.001**
V55(%)	0.457	**0.000**	0.453	**0.000**
V60(%)	0.393	**0.002**	0.371	**0.004**
V65(%)	0.270	**0.037**	0.261	**0.044**
V70(%)	0.249	0.055	0.199	0.128
D_{mean}(Gy)	0.325	**0.011**	0.269	**0.036**
H-D_{mean} (Gy)	0.300	**0.019**	0.314	**0.014**

H-D_{mean} is the highest mean dose between left and right parotid.

Table S10. Spearman's Rho values between ADC/D_t variations and dose-volume data of both glands at 10th fraction.

Variable	ADC(%)		D_t (%)	
	**Spearman's Rho**	**p-value**	**Spearman's Rho**	**p-value**
V20(%)	0.041	0.762	0.004	0.976
V25(%)	0.169	0.214	0.132	0.331
V30(%)	0.317	**0.017**	0.289	**0.031**
V35(%)	0.362	**0.006**	0.353	**0.008**
V40(%)	0.378	**0.004**	0.378	**0.004**
V45(%)	0.381	**0.004**	0.409	**0.002**
V50(%)	0.369	**0.005**	0.417	**0.001**
V55(%)	0.397	**0.002**	0.430	**0.001**
V60(%)	0.295	**0.028**	0.347	**0.009**
V65(%)	0.191	0.159	0.244	0.070
V70(%)	0.124	0.363	0.139	0.308
D_{mean}(Gy)	0.372	**0.003**	0.370	**0.004**
$D_{mean, highest}$ (Gy)	0.349	**0.006**	0.400	**0.002**

Statistically significant p-values are ***bold.***

Table S11. Selected (categorical) predictor for Xerostomia Grade ≥ 2 12 months after radiotherapy.

**N stage**	**XER_{12} < 2**	**XER_{12} = 2**	**P-value (Chi-squared)**
N0	6	1	0.034
N1	17	3	
N2	14	15	
N3	5	2	

Table S12. Spearman's coefficient Rho between selected (continuous) variables.

	K^{trans} P10	***v_e P25***	***V65(%)***	***D_{mean}***	***$D_{mean,SMG}$***	***XQ-Int_{mid}***
K^{trans} P10	1.00					
v_e P25	0.62	1.00				
**V65**	-0.25	-0.13	1.00			
**D_{mean}**	-0.19	0.03	0.68	1.00		

$D_{mean,SMG}$	-0.31	-0.34	0.39	0.24	1.00	
**XQ-Int**$_{mid}$	-0.05	-0.08	0.33	0.32	0.32	1.00

Table S13. Comparison between prediction accuracies of the models. P-values refer to the mid-p-value McNemar test.

	Models based on variables at baseline							Models based on variables at baseline and during RT								
	**1**	**2**	**3**	**4**	**5**	**6**	**7**	**8**	**9**	**10**	**11**	**12**	**13**	**14**	**15**	**16**
**1**	1.00	0.30	0.06	0.73	0.86	0.63	0.61	0.09	0.50	0.05	0.51	**0.02**	0.09	0.47	0.56	0.34
**2**	0.30	1.00	**0.01**	0.12	0.41	0.60	0.12	**0.00**	0.75	**0.01**	0.09	**0.00**	**0.01**	0.71	0.09	**0.04**
**3**	0.06	**0.01**	1.00	0.10	**0.04**	**0.04**	0.21	0.86	0.05	1.00	0.38	0.79	1.00	**0.03**	0.21	0.36
**4**	0.73	0.12	0.10	1.00	0.61	0.34	0.86	0.15	0.36	0.18	0.71	0.08	0.20	0.31	0.85	0.56
**5**	0.86	0.41	**0.04**	0.61	1.00	0.72	0.30	0.07	0.67	0.06	0.41	**0.04**	0.05	0.61	0.46	0.23
**6**	0.63	0.60	**0.04**	0.34	0.72	1.00	0.26	**0.02**	0.88	0.05	0.27	**0.03**	**0.04**	0.87	0.32	0.18
**7**	0.61	0.12	0.21	0.86	0.30	0.26	1.00	0.24	0.31	0.26	0.86	0.17	0.19	0.22	1.00	0.63
**8**	0.09	**0.00**	0.86	0.15	0.07	**0.02**	0.24	1.00	**0.00**	0.82	0.12	1.00	0.84	**0.01**	0.17	0.34
**9**	0.50	0.75	0.05	0.36	0.67	0.88	0.31	**0.00**	1.00	**0.02**	0.11	**0.01**	**0.02**	1.00	0.24	0.13
**10**	0.05	**0.01**	1.00	0.18	0.06	0.05	0.26	0.82	**0.02**	1.00	0.29	0.75	1.00	**0.01**	0.09	0.30
**11**	0.51	0.09	0.38	0.71	0.41	0.27	0.86	0.12	0.11	0.29	1.00	0.23	0.33	0.15	0.83	0.84
**12**	**0.02**	**0.00**	0.79	0.08	**0.04**	**0.03**	0.17	1.00	0.01	0.75	0.23	1.00	0.80	**0.01**	0.10	0.18
**13**	0.09	**0.01**	1.00	0.20	0.05	**0.04**	0.19	0.84	**0.02**	1.00	0.33	0.80	1.00	**0.02**	0.23	0.33
**14**	0.47	0.71	**0.03**	0.31	0.61	0.87	0.22	**0.01**	1.00	**0.01**	0.15	**0.01**	**0.02**	1.00	0.22	0.09
**15**	0.56	0.09	0.21	0.85	0.46	0.32	1.00	0.17	0.24	0.09	0.83	0.10	0.23	0.22	1.00	0.63
**16**	0.34	**0.04**	0.36	0.56	0.23	0.18	0.63	0.34	0.13	0.30	0.84	0.18	0.33	0.09	0.63	1.00

Statistically significant p-values are ***bold.***

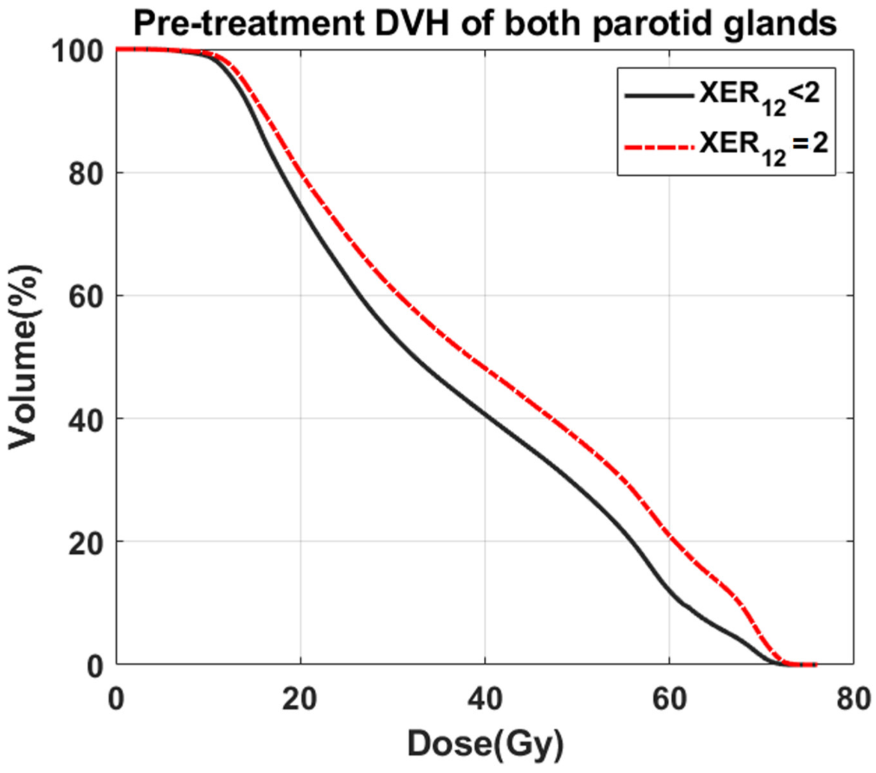

Figure S1. The average of the distribution of cumulative dose–volume histograms (DVHs) of both parotids, for patients with salivary gland toxicity at 12 months after radiotherapy (XER$_{12}$) < grade 2 (solid line) and = grade 2 (dashed line).

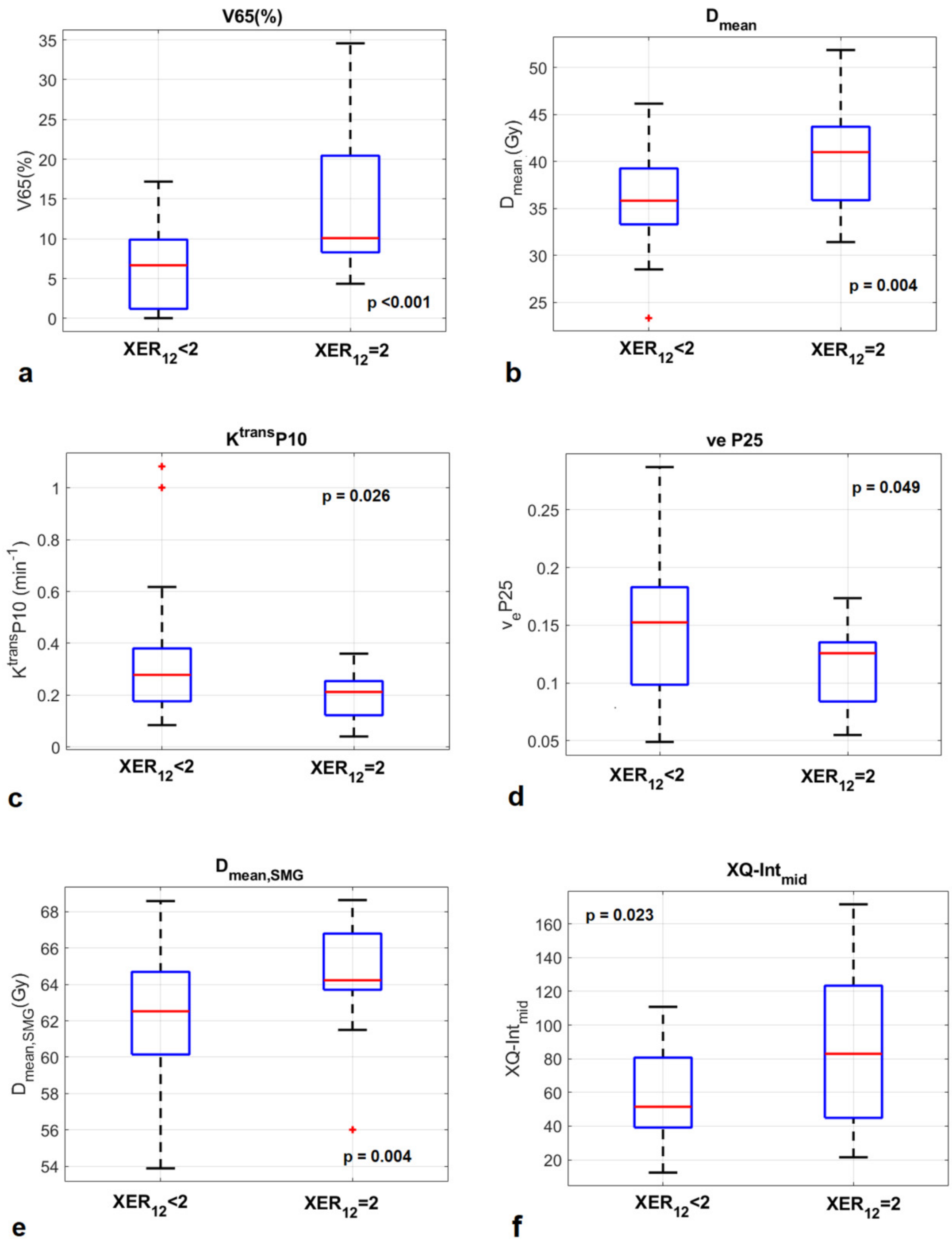

Figure S2. Box-and-whisker plots of the most significant predictors for XER_{12} = grade 2. P values for the Mann-Whitney test are reported in each plot.

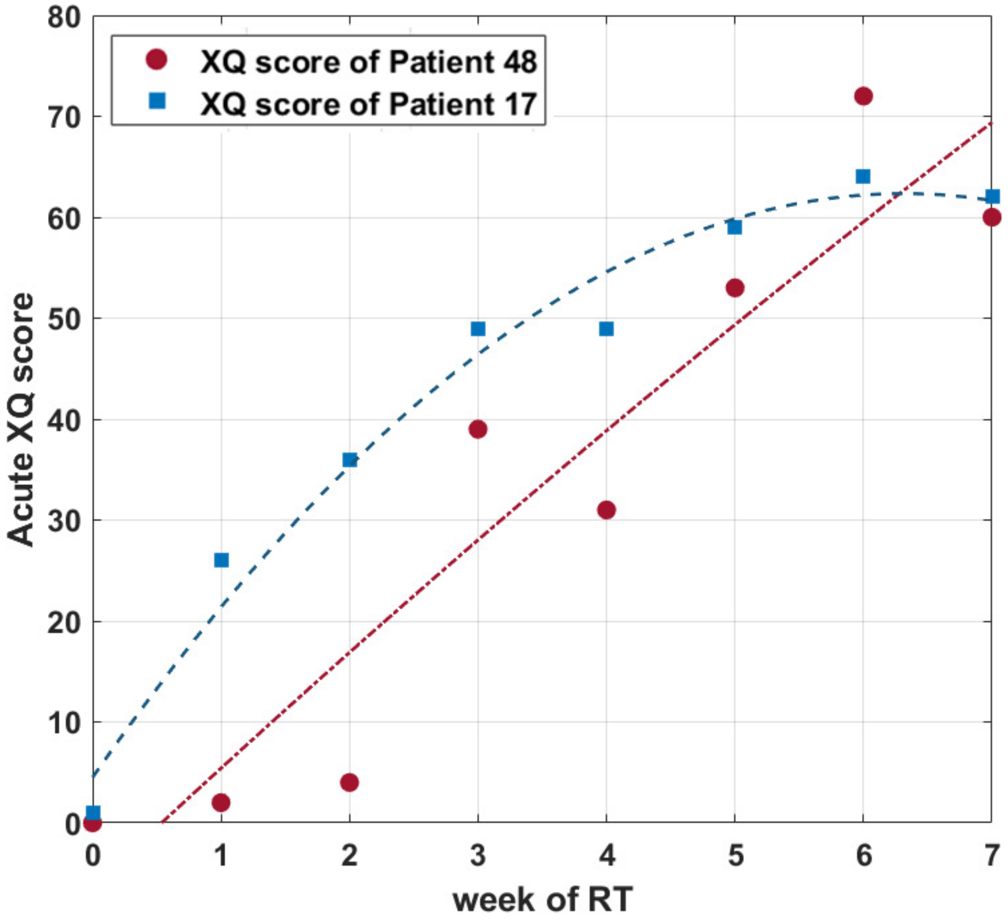

Figure S3. Plots of acute XQ-score versus week of RT in two representative patients. Patient 48, who developed grade 1 XER_{12} showed a lower curve of acute XQ scores versus treatment weeks than Patient 17, who experienced grade 2 XER_{12} ($XQ\text{-}Int_{mid}$ = 33.2 versus 82.9, XQ-Grad1 = 11.6 versus 15.4 and XQ-Grad2= 10.7 versus 6.7, respectively). These data refer to the same patients showed in Figure 2 of the manuscript.
